# Distribution Pattern and Assembly Process of Fungal Communities Along Altitude Gradient in Sediments of the Yellow River Basin

**DOI:** 10.3390/jof11030214

**Published:** 2025-03-11

**Authors:** Kang Fang, Guoce Xu, Xin Chen, Jing Li, Yuting Cheng, Yifan Cheng

**Affiliations:** 1 State Key Laboratory of Water Engineering Ecology and Environment in Arid Area, Xi’an University of Technology, Xi’an 710048, China; kangjie5065@163.com (K.F.); cx852329@163.com (X.C.); lijing8615@163.com (J.L.); 15369869675@163.com (Y.C.); 2 Geology and Environment, Xi’an University of Science and Technology, Xi’an 710048, China; chengyutingstar@163.com

**Keywords:** altitudinal gradient, fungal community, distribution pattern, assembly process, Yellow River basin

## Abstract

Microorganisms have a profound impact on the stability and ecological health of aquatic environments. Fungi, as important components of river ecosystems, play critical roles as decomposers and symbionts. A comprehensive understanding of the mechanisms underlying fungal community assembly is essential for the effective conservation and management of river ecosystems. However, the distribution patterns and assembly process of fungal communities along elevation gradients in river sediments remain poorly understood. In this study, ITS amplicon sequencing, a neutral community model, and a null model were employed to analyze the distribution patterns and assembly processes of fungal communities in sediments along the altitudinal gradient of the Yellow River. The results indicated that Ascomycota (47.79%) and Basidiomycota (15.68%) were identified as the dominant phyla in the sediments, collectively accounting for 63.47% of the total relative abundance of the community. In the three different altitudinal gradients, the fungal community diversity (Shannon) showed a gradually decreasing trend with increasing altitude. The co-line networks of fungal communities exhibited positive interactions and had more complex and compact networks in the sediments of the Tibetan Plateau area (YRA). Environmental factors in the sediments played an important role in shaping the structure of fungal communities, with lead (Pb), total nitrogen (TN), silt, and total organic carbon (TOC) being the main factors driving changes in community structure, contributing 15.5%, 12.3%, 10.7%, and 10.2%, respectively. In the community assembly process, deterministic processes were found to dominate, with homogenizing selection contributing the most (69.66%). These research results help us understand the distribution patterns of fungal communities along altitudinal gradients and the mechanisms of community assembly, and also provide a scientific basis for biodiversity conservation and the rational use of biological resources.

## 1. Introduction

Rivers are key components of the hydrological cycle, providing indispensable water resources for industrial and agricultural activities [1]. However, aquatic ecosystems have been severely impacted by the intensification of human activities and the disruptions caused by global environmental changes [2,3]. Microorganisms serve as decomposers in aquatic ecosystems, playing a crucial role in maintaining structural and functional stability [3,4]. Fungi represent a significant component of microbial communities, comprising more than 140,000 species, and exhibit the highest diversity within the eukaryotic domain [5]. Fungal communities are widely distributed across diverse habitats, including freshwater, marine, and terrestrial environments [4,6,7]. Fungal communities can act as decomposers and facilitators in the cycling of organic matter, functioning both as parasites/pathogens and as symbionts with higher trophic organisms to participate in material cycling [8,9,10]. As fungal communities participate in diverse ecological processes, variations in their composition can provide valuable insights for assessing river water quality and ecosystem health [11,12].

In order to adapt to the stresses brought about by environmental change, microorganisms often develop unique community structures under the influence of a variety of factors [13]. The community assembly process of microorganisms refers to the process in which microorganisms interact through different ecological and evolutionary processes under specific environmental conditions to ultimately form a stable community [14]. The community assembly process has been extensively applied in microbial research [1,15,16,17,18,19]. Community assembly process research seeks to distinguish and quantify ecological processes, using statistical analysis, model inference, and network analysis methods to explain microbial community responses to disturbances, which helps us to understand the mechanisms maintaining microbial community stability [20]. Deterministic and stochastic processes are two important theories to explain species coexistence. Both of them jointly affect the assembly process of microbial communities [16,21,22], but the relative importance of these two mechanisms in the community assembly process varies among different ecosystems. The assembly mechanisms of fungal communities involve multiple ecological processes, including selection, dispersal, speciation, and drift [23]. In recent years, driven by advancements in high-throughput sequencing technologies and bioinformatics, significant progress has been made in the study of fungal community assembly mechanisms. It is not clear whether the community distribution pattern and community building mechanism of fungal communities in sediments of the Yellow River Basin differ from those of other rivers under differences in elevation gradients.

The Yellow River is the second longest river in China, and for thousands of years it has been the fulcrum of the development and progress of Chinese civilization. The Yellow River is famous for its high sediment content, and with severe sedimentation and unpredictable channel changes, the ecological environment within the basin is highly sensitive and fragile [24,25]. The Yellow River Basin is excellent to study microbial communities due to its complex geomorphology and diverse sedimentary environments. Some studies have been conducted on the Yellow River Basin, showing that there are differences in bacteria and fungi in the sediments of the Yellow River oxbow lakes at three successional stages [24]; the dominant bacterial phyla in the sediments of the Yellow River are Proteobacteria, Firmicutes, and Bacteroidetes [26]; water temperature (WT), electrical conductivity (EC) and NH_4_^+^-N are factors contributing to the differences in microbial structure in Yellow River water and the sediment column in the Jinan section [27]. Although several studies on microbial communities in the Yellow River Basin have been conducted, most have focused on specific sections or tributaries, covering a relatively limited scope. There are few reports on the distribution patterns and assembly mechanisms of fungal communities in sediments across the entire basin.

In this study, ITS high-throughput sequencing was employed to investigate fungal community distribution patterns and assembly mechanisms in sediments across elevational gradients within the Yellow River Basin. The study aims to determine the following: (1) the characteristics of fungal communities in sediments at different altitudinal gradients; (2) distribution patterns of fungal communities in sediments along elevational gradients; and (3) assembly processes of fungal communities in sediments.

## 2. Materials and Methods

### 2.1. Study Area

**Figure 1:**
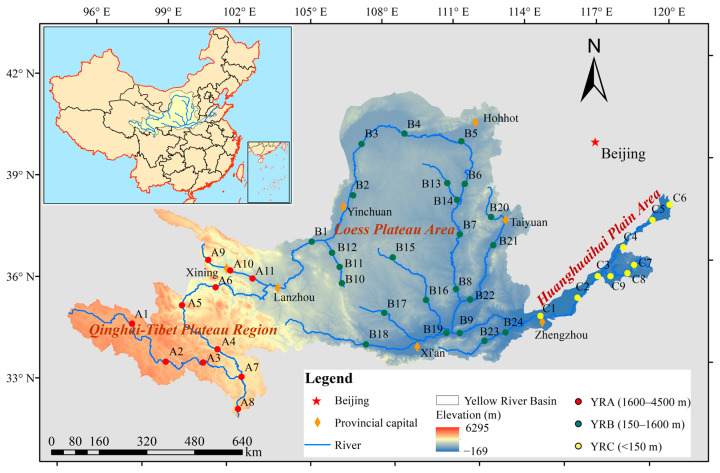
Schematic diagram of the Yellow River Basin and sampling points.

The Yellow River is China’s second-longest watercourse (5464 km), originating from the Yoguzonglei Basin in the northern foothills of the Ba Yan Ka La Mountains on the Qinghai–Tibet Plateau, and crossing the three terraces from west to east, it flows through a number of geomorphologic units before being injected into the Bohai Sea in Kenli County, Shandong Province. The basin area is 795,000 km^2^ (32°10′~41°50′ N, 95°53′~119°05′ E), and belongs to a typical transition zone between a monsoon and continental climate [28], with an annual average temperature ranging from −4 to 14 °C. Precipitation in the basin is unevenly distributed in time and space, mainly concentrated in July and August, with amounts ranging from 140 mm per year in the northern part of the basin to 1100 mm per year in the eastern part and an average annual precipitation of 495.6 mm [29]. Forty-four sampling sites were arranged in the Yellow River Basin (Figure 1), and the sampling sites were categorized according to the elevation gradient: the YRA group (1600–4500 m, Qinghai–Tibetan Plateau area), YRB group (150–1600 m, Loess Plateau area), and YRC group (<150 m, Yellow-Huai-Hai Plain area).

### 2.2. Sample Collection and Analysis

Surface sediment samples (500 g) were collected from the main stem and major tributaries of the Yellow River Basin during July and August 2022 using a grab sediment sampler. Following collection, a portion of the samples was transferred into 10 mL sterile centrifuge tubes for fungal community composition analysis, while the remainder was preserved in sterile sampling bags for subsequent physicochemical characterization. The coordinates and elevation of the sampling sites were recorded using a GPS locator. A fully automated intermittent chemistry analyzer (SmartChem 200, AMS Allinace, Rome, Italy) was used for the determination of total nitrogen (TN), ammonia nitrogen (NH_4_^+^-N), nitrate nitrogen (NO_3_^−^-N), and total phosphorus (TP) in sediments. A Malvern laser particle sizer (Malvern Instruments, Malvern, UK) was used to determine sediment particle size. An organic carbon analyzer (multi N/C^®^ 3100, Jena, Germany) was used to determine the total organic carbon (TOC) content in sediments. Following acid digestion, the concentrations of heavy metals (Cr, Ni, Cu, Zn, As, Cd, Pb) were measured by inductively coupled plasma mass spectrometry (iCAP Q, Thermo Fisher Scientific, Waltham, MA, USA).

### 2.3. High-Throughput Sequencing

DNA from the samples was extracted following the guidelines provided by the Soil DNA Rapid Extraction Kit (MP Biomedicals, Santa Ana, CA, USA), followed by qualitative assessment through 1% agarose gel electrophoresis. The amplification of the fungal ITS region was performed using the universal primers ITS1F_ITS2R [9,25]. The resulting PCR products were then recovered from 2% agarose gel and purified with a DNA gel extraction kit [25,30]. Additional methodological details are available in the Supplementary Materials. DNA extraction and sequencing were entrusted to Shenzhen Microman Technology Group Co. (Shenzhen, China) (www.bioincloud.tech).

### 2.4. Data Statistics and Microbial Analysis

Statistical analyses were conducted using SPSS 23.0 (IBM Corp.) and Microsoft Excel 2010, with significance determined through one-way analysis of variance (ANOVA) followed by Tukey’s post hoc tests (*p* < 0.05). Community alpha-diversity indices (Shannon and Simpson) were calculated alongside non-metric multidimensional scaling (NMDS) and analysis of similarity (ANOSIM) using the Vegan package (v2.6-4) in R (v4.2.1). Distance–decay relationships were quantified through geospatial analysis implemented with the vegan (v2.6-4) and geosphere (v1.5-18) packages. β-diversity patterns were assessed based on Bray–Curtis dissimilarity matrices using the NST package (v1.2.1). Neutral community modeling was performed using the “Hmisc” package. Redundancy analysis was performed using Canoco.5 to explore the effect of environmental factors on fungal communities. Visualization of co-occurrence networks was implemented in Gephi (0.10.1). The location map of the study area and the sampling site diagram were drawn using ArcMap 10.2.

## 3. Results

### 3.1. Species Composition and Diversity of Fungal Communities

**Figure 2:**
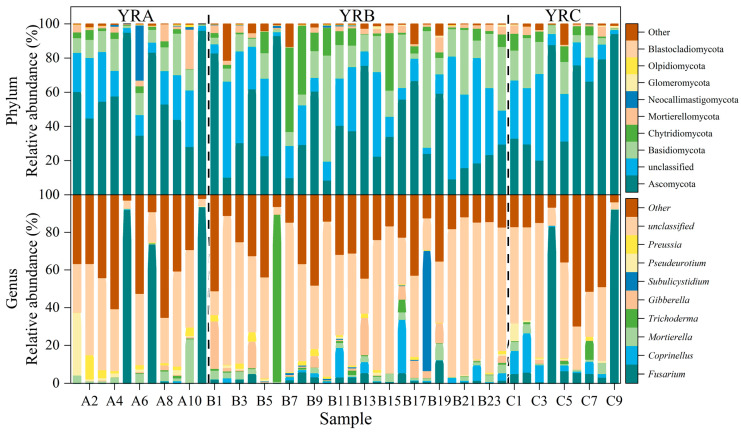
Characterization of fungal communities in Yellow River sediments.

High-throughput ITS sequencing was conducted on fungal communities in sedimentary samples, with the relative abundance distributions of the top ten phyla and genera presented in Figure 2. Through taxonomic abundance analysis, 23 phyla and 674 genera were identified through species abundance analysis and annotation. In the taxonomic hierarchy, microbial groups with lower abundance rankings were classified as “other”. The results showed that 0.28% and 28.23% of fungi were classified as “other” at the phylum and genus levels, respectively. In addition, the number of ASVs (Amplicon Sequence Variants) unique to fungal communities in YRA, YRB, and YRC were 2930, 5991, and 1670, respectively, with 280 ASVs shared among the three altitudinal regions (Figure S3).

The fungal communities were composed of Ascomycota (47.79%), unclassified (23.54%), Basidiomycota (15.68%), Chytridiomycota (6.47%), Mortierellomycota (2.68%), and Neocallimastigomycota (1.24%) (Figure 2). Ascomycota and Basidiomycota were the first and second dominant phyla in sediments, respectively, with Ascomycota having abundances of 58.53%, 38.64%, and 57.61% in YRA, YRB, and YRC, respectively; Basidiomycota had abundances of 18.25%, 26.89%, and 21.08% in YRA, YRB, and YRC, respectively. In addition, the abundance of Chytridiomycota in B7, B8, and B15 of the YRB group was significantly higher than in other sampling sites, with abundances of 53.99%, 39.17%, and 33.93%, respectively; in the YRA group, the abundance of Neocallimastigomycota was significantly higher than in other sampling sites, with an abundance as high as 47.47%.

Fungal community composition at the genus level was characterized by unclassified taxa (43.65%), *Fusarium* (12.10%), *Coprinellus* (2.98%), *Mortierella* (2.81%), *Trichoderma* (2.79%), and *Gibberella* (2.62%) in descending order of relative abundance. *Fusarium* was the dominant genus in the fungal community, with abundances of 23.88%, 2.56%, and 23.13% in YRA, YRB, and YRC, respectively; the abundance in YRA and YRC was significantly higher than that in YRB (*p* < 0.05). In addition, the relative abundance of *Fusarium* was higher in A5, A7, A11, C4, and C9 than in other sample sites (*p* < 0.05), whereas the abundance of *Trichoderma* was the highest in sample site B6, with an abundance of 88.69%.

**Figure 3:**
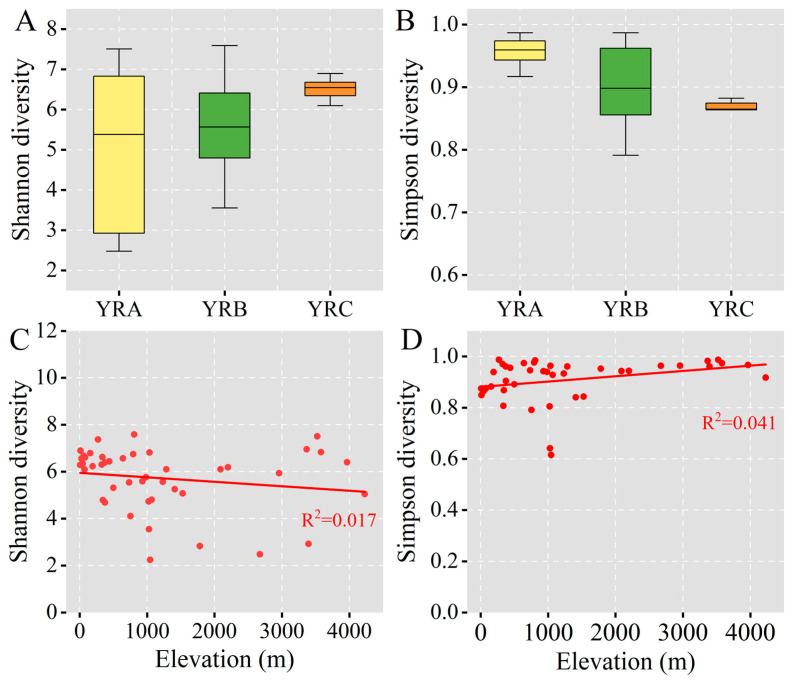
α-diversity of fungal community. (**A**) Distribution characteristics of fungal community Shannon diversity along the altitudinal gradient. (**B**) Distribution characteristics of fungal community Simpson diversity along the altitudinal gradient. (**C**) Variation characteristics of fungal community Shannon diversity with altitude. (**D**) Variation characteristics of fungal community Simpson diversity with altitude.

A gradual decline in the Shannon diversity index was observed with increasing altitude (Figure 3), whereas the Simpson diversity index demonstrated an inverse pattern in its correlation with elevation. The Shannon diversity index was recorded at 5.38 in YRA. In comparison to YRA, elevations YRB and YRC exhibited increases of 3.35% and 21.56%, respectively.

### 3.2. Biogeographical Analysis of Fungal Communities

**Figure 4:**
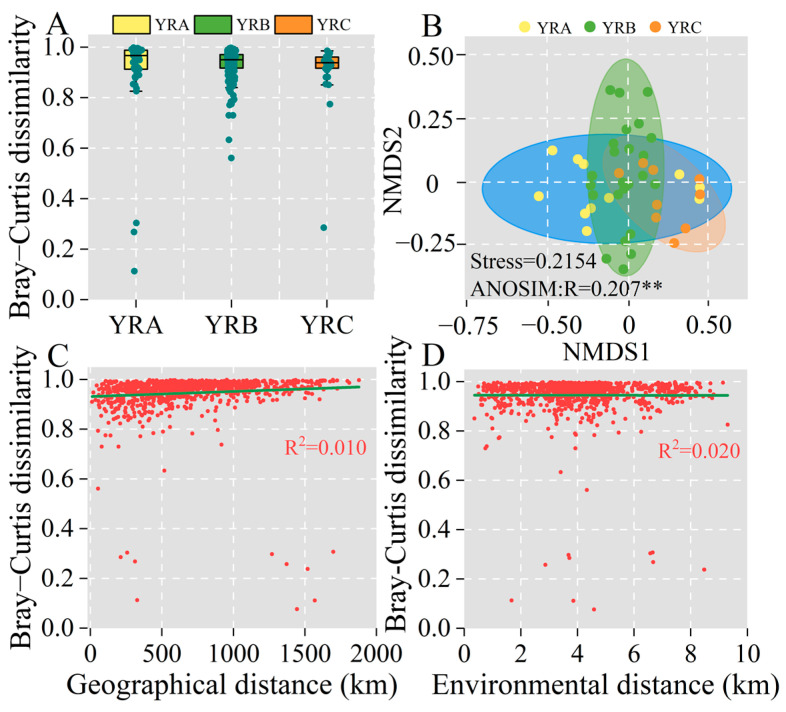
β-diversity of fungal community. (**A**) Spatial characterization of Bray–Curtis differences in fungal communities. (**B**) Non-metric multidimensional scaling analysis (NMDS) of elevation gradients in fungal communities. (**C**) The variation characteristics of fungal community Bray–Curtis dissimilarity with increasing geographical distance. (**D**) The variation characteristics of fungal community Bray–Curtis dissimilarity with increasing environmental distance. Note: ** indicates *p* < 0.01.

Differences in fungal community structure in sediments from different elevation gradients are shown in Figure 4, with Bray–Curtis dissimilarity of 0.96, 0.95, and 0.93 for YRA, YRB, and YRC, respectively. To explore the potential driving factors of fungal community variation, we conducted biogeographical analysis. The distance–decay relationship is a well-known biogeographical model that explores the relationship between regional geographical distance (stochastic process) and local environmental distance (deterministic process) and community differences [31]. Among all sampling sites, the differences in fungal communities significantly increased with increasing geographical distance (*p* < 0.01) (Figure 4C). Across altitudinal gradients, differences in fungal communities in the YRA group had the strongest attenuation effect with geographic distance across the altitudinal gradient compared to YRB and YRC. In addition, the environmental distance attenuation effect of fungal communities in sediments at the three altitudinal gradients was stronger than the geographical distance (Figure 3C,D), indicating that the distribution pattern of fungal communities in sediments of the Yellow River Basin was more influenced by environmental factors.

### 3.3. Characteristics of Fungal Community Co-Occurrence Networks

**Figure 5:**
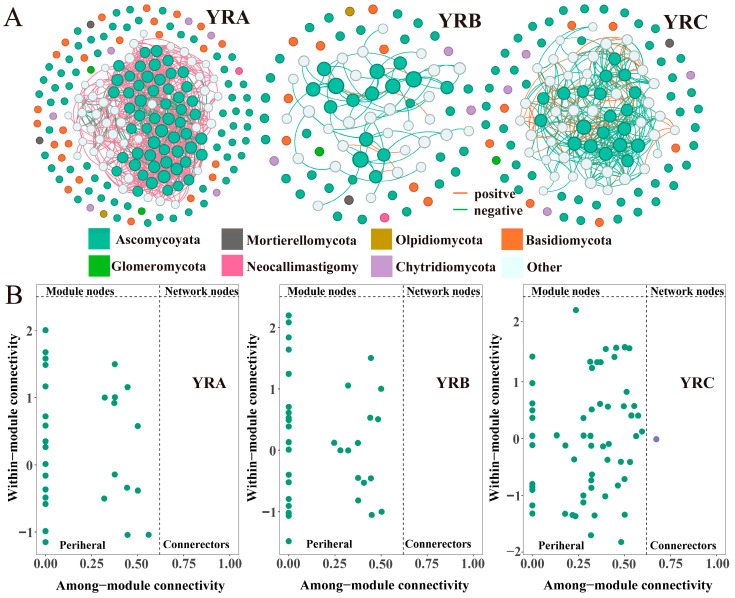
Co-linear network analysis of fungal communities. (**A**) Co-linear network of fungal communities in sediments at different elevation gradients. (**B**) Key species analysis.

A co-occurrence network analysis was conducted to examine fungal communities in sediments of the Yellow River Basin across three distinct altitudinal gradients (Figure 5). The results demonstrated that interspecific connectivity within fungal communities was predominantly characterized by positive correlations across all altitudinal gradients, indicating that synergistic interactions prevailed over competitive relationships. Higher values of nodes (61) and average degree (10.75), combined with a lower average path length (2.27), were observed in YRA (Table 1). The number of edges in the co-occurrence network of fungal communities in YRA sediments was 327, significantly higher than that in YRB (54) and YRC (66). This suggests that fungal communities in YRA sediments exhibit greater network complexity and connectivity and are more concentrated than YRB and YRC.

Generally, network nodes, module nodes, and connectors are considered keystone species in building communities and may play important roles in maintaining community structure [15]. If these taxa are removed, modules and networks may collapse. In our study, fungal communities in sediments at different altitudinal gradients in the Yellow River Basin did not have module nodes and network nodes, with only one connector present in YRC (Figure 5B).

### 3.4. Analysis of Fungal Community Assembly Processes

**Figure 6:**
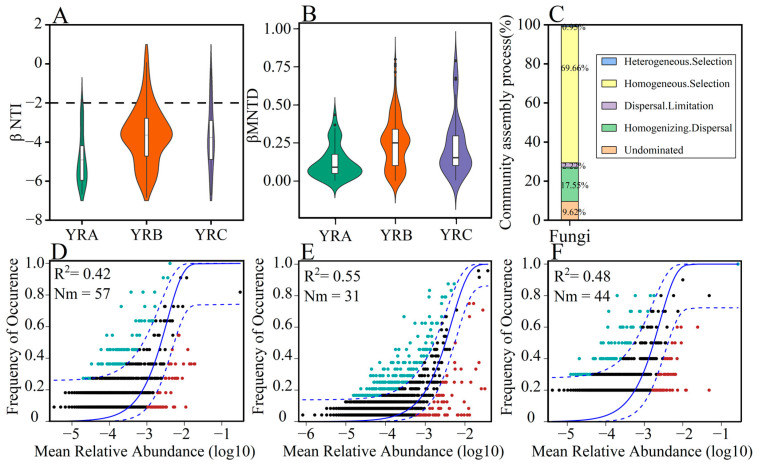
Fungal community assembly processes. (**A**) Changes in fungal community βNTI with altitudinal gradient. (**B**) Changes in fungal community βMNTD with altitudinal gradient. (**C**) The role of different ecological processes in assembling fungal communities. (**D**) Neutral community model fitting of fungal communities in YRA. (**E**) Neutral community model fitting of fungal communities in YRB. (**F**) Neutral community model fitting of fungal communities in YRC. Note: Green and red dots represent those ASVs that occur more frequently and less frequently than predicted by the model, respectively. The solid blue line shows the best-fit result of the neutral community model, while the dashed blue line marks the 95% confidence interval. Nm indicates the estimated migration rate, while R^2^ reflects the degree of model fit.

To further quantify the relative contributions of stochastic versus deterministic processes in microbial community assembly, the phylogenetic normalized stochasticity ratio (pNST) was determined through null model analysis (Figure 6). The βNTI results indicated that deterministic processes played a dominant role in the assembly of fungal communities, accounting for 88.56%. During the assembly process, homogenizing selection accounted for the highest proportion (69.66%) in the assembly of fungal communities. In addition, homogenizing dispersal also made a significant contribution to the assembly of fungal communities, accounting for 17.55%. The migration rate (Nm) estimated by the neutral model reflects the dispersal ability of the species, with higher values of Nm resulting in higher migration rates [32]. The Nm value in YRA sediments was the highest (Figure 6D), indicating that the fungal communities in YRA had a higher migration rate.

### 3.5. Analysis of Factors Influencing Fungal Community Differences

**Figure 7:**
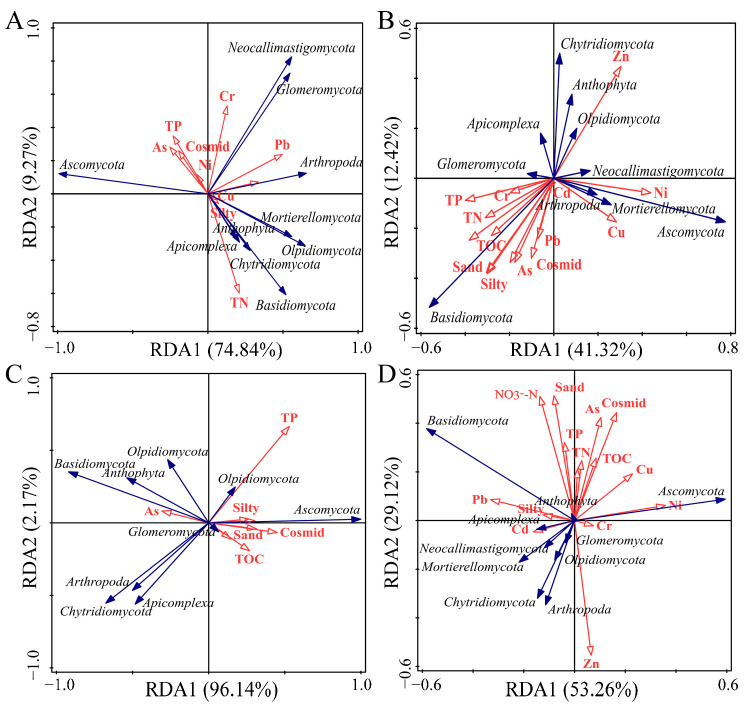
Redundancy analysis of fungal communities in sediments and environmental factors. (**A**) YRA group. (**B**) YRB group. (**C**) YRC group. (**D**) Entire basin.

The physicochemical properties of the sediment were measured, and the results are presented in Figures S1 and S2. Redundancy analysis (RDA) was performed to assess the influence of sediment environmental factors on fungal community variations (Figure 7 and Table 2). The first RDA axis accounted for the majority of fungal community variation, capturing 41% of the total explained variance. Significant explained quantities in the YRA group were Pb (19.4%), Ni (24.0%), TN (17.4%), and silt (11.6%); significant explained quantities in the YRB group were Ni (13.9%), sand (14.4%), and TN (10.4%); significant explained quantities in the YRC group were TP (27.9%), Consimd (24.0%), As (12%), and NO_3_^−^-N (11.6%); significant explained quantities in the Yellow River Basin group were TOC (10.2%), Pb (15.5%), silt (10.7%), and TN (12.3%). Therefore, carbon, nitrogen, sediment particle composition, and heavy metal content are the primary factors influencing the differences in fungal communities within the sediments of the Yellow River Basin.

Significant negative correlations between Pb and Ascomycota were observed (*p* < 0.05), with spatial heterogeneity across altitudinal gradients: a negative association in YRA (*p* < 0.05), a positive correlation in YRB (*p* < 0.05), and a non-significant relationship in YRC. Total organic carbon (TOC) exhibited basin-scale positive correlations with Ascomycota (*p* < 0.05), though gradient-specific variations emerged: non-significant in YRA, negative in YRB (*p* < 0.05), and positive in YRC (*p* < 0.05). Silt content demonstrated basin-wide negative correlations with Ascomycota (*p* < 0.05), showing consistent negativity in YRA/YRB (*p* < 0.05) but positivity in YRC (*p* < 0.05). Regarding Basidiomycota, abundance was positively associated with Pb at the basin scale (*p* < 0.05), showing significant correlations in YRA/YRB (*p* < 0.05) but no significance in YRC. Notably, TOC and silt content showed contrasting spatial patterns with Basidiomycota: basin-scale positive correlations (*p* < 0.05) existed alongside non-significant (YRA), positive (YRB), and negative (YRC) regional associations (*p* < 0.05).

## 4. Discussion

### 4.1. Fungal Community Structure and Diversity at Different Altitudinal Gradients

The characteristics of river microbial communities are one of the key bases for evaluating the health of ecological environments, and the distribution patterns and diversity of microbial communities directly affect the stability of the structure and function of river ecosystems [1,33]. In this study, we found that fungal community diversity decreased with increasing altitude. It has been found that microbial diversity at high altitude is negatively correlated with altitude [15], and our results are consistent with this finding. This may be due to the differences in sedimentary environmental conditions at different altitudinal gradients leading to differences in fungal community composition and diversity. Secondly, the high content of heavy metals in the sediments of the YRA area led to a decrease in fungal community diversity.

**Figure 8:**
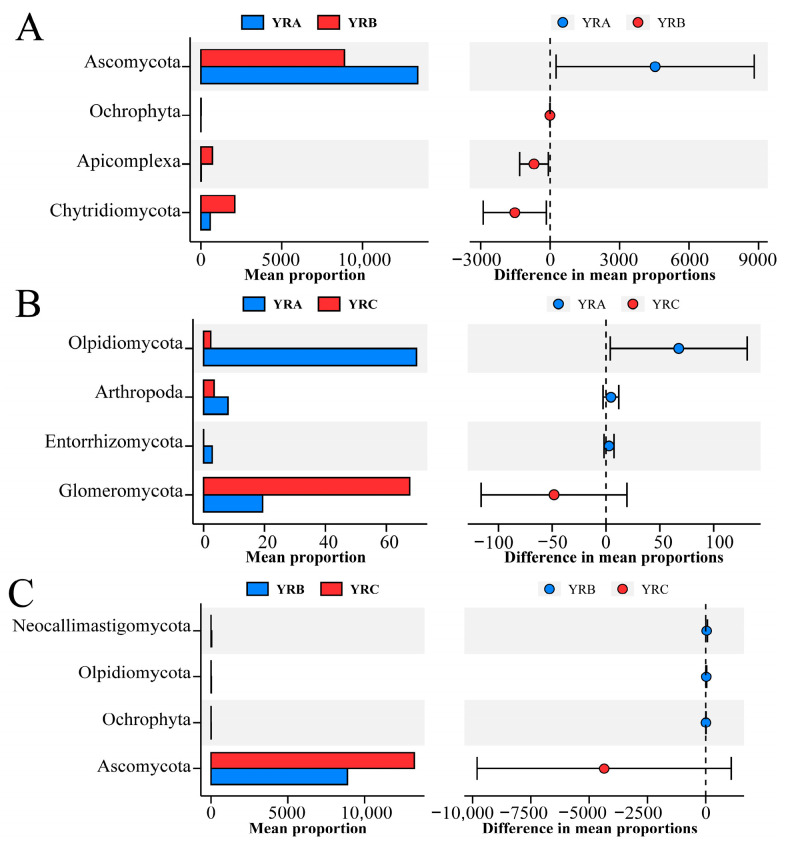
Differences in fungal community composition in sediments from the Yellow River Basin. (**A**) Differences in YRA and YRB communities. (**B**) Differences between YRA and YRC communities. (**C**) Difference between YRB and YRC communities.

Similarly to previous studies, Ascomycota and Basidiomycota are the dominant phyla of fungal communities in the sediments of the Yellow River Basin [34]. We used Stamp analysis to explore the significantly different taxa in sediments at different altitudinal gradients (Figure 8), which are often closely related to river environmental factors. The Yellow River is a typical high-silt river, and its microbial community structure is highly complex and variable due to human activities and climate change. While existing research has predominantly examined microbial community structure and diversity in the Yellow River ecosystem, there remains a paucity of investigations specifically addressing spatial distribution patterns and assembly mechanisms of sedimentary fungal communities.

Analytical results reveal that Ascomycota and Basidiomycota constitute the predominant phyla within fungal communities in the sedimentary environments of the Yellow River Basin. Ascomycota was observed to dominate with a relative abundance of 47.79%, followed by Basidiomycota at 15.68%. These observations align with previous findings from the Wuding River ecosystem, where Ascomycota has been reported as the dominant phylum in benthic fungal communities, exhibiting relative abundances of 38.72% and 19.47% during non-flood and flood seasons, respectively [35]. In contrast, Chytridiomycota has been documented as the predominant group during dry seasons in the Mekong River, whereas Ascomycota and Basidiomycota prevail in rainy seasons [9]. Similarly, Ascomycota (60%) and Basidiomycota (32%) have been identified as the dominant components of fungal assemblages in central Finnish lotic systems [36]. The abundance of Ascomycota was significantly higher in the YRA and YRC regions than in the YRB region. This is mainly because the high concentration of nutrients in the YRA region promotes the reproduction of Ascomycota, while the low-flow aquatic environment in the YRC region is conducive to the colonization and growth of Ascomycota in sediments. Basidiomycota are important decomposers that can break down organic matter in river sediments, such as plant residues and lignocellulose, converting complex organic compounds into simpler inorganic substances, thereby promoting the cycling of carbon and nitrogen [37]. The abundance of Chytridiomycota in B7, B8, and B15 of the YRB group was significantly higher than that of other sample sites, with its abundance being 53.99%, 39.17%, and 33.93%, respectively, which may be due to the entry of residual straw from the crops into the riverine environment in these sample sites.

In addition, 6.47%, 2.68%, and 1.24% of the fungal communities were identified as Chytridiomycota, Mortierellomycota, and Neocallimastigomycota, respectively. However, the relative abundance of these fungi varied among all samples, and some were not detected at certain sampling sites; these low-frequency fungi may play specific ecological roles in the sediments of the Yellow River Basin [38]. At the genus level, dominant taxa were identified as *Fusarium*, *Coprinellus*, *Mortierella*, *Trichoderma*, *Gibberella*, and *Subulicystidium*. Interestingly, these genera are predominantly associated with terrestrial ecosystems, particularly inhabiting soil matrices and decomposing plant detritus. These fungi are usually widely distributed in terrestrial ecosystems, especially in soil and plant residues. Their high abundance in the sediments of the Yellow River is related to the serious soil erosion in the Yellow River Basin. The Yellow River Basin is one of the most severely soil-eroded areas in the world, with the sediment discharge of the Yellow River reaching 1.59 × 10^8^ t/yr [39]. During the process of soil erosion, fungal communities from soil and plant residues enter the river environment and colonize the sediments. Although these fungi are not typical aquatic fungi, their presence has certain ecological significance for the aquatic ecosystem.

### 4.2. Assembly Processes of Fungal Communities in Sediments

The mechanisms underlying biodiversity formation and maintenance, specifically the process of community assembly, have long constituted a central focus in ecological research. Furthermore, investigations into microbial community assembly processes currently represent a prominent research frontier in microbial ecology [40,41]. Neutral models and standardized stochastic rates have been recognized as critical analytical tools in ecological research, enabling effective quantification of the relative contributions made by deterministic and stochastic factors during community assembly processes [42,43]. The mechanism of riverine microbial community formation may be influenced by a variety of factors, among which changes in environmental factors are an important driver, considering that differences in birth, apoptosis, community evolution, and species formation in the aquatic environment shape different types of microbial communities [44,45,46]. The assembly of microbial communities involves both deterministic and stochastic processes [22,47,48], and these two mechanisms complement each other, but at the same time there are interactions and constraints.

This study showed that homogenizing selection accounted for the highest proportion (69.66%) in the assembly of fungal communities in sediments. Homogenizing selection is the environmental selection effect formed by the interaction between similar abiotic environments in space and time and biota, which may lead to convergence in microbial community composition [49,50,51]. Our study found that deterministic processes (especially homogenizing selection) play a dominant role in the assembly of riverine fungal communities. This finding is consistent with some similar studies, but also has differences. For example, in the study of the Three Gorges Reservoir, homogenizing selection was also considered as the main driving force for microbial community assembly [52]. However, in the study of the Shenzhen River estuary continuum, the assembly of bacterial communities was mainly affected by homogeneous selection and dispersal limitation [53]. Dispersal limitation is a key ecological process in the assembly of fungal communities. In mining-affected rivers, the proportion of fungal community assembly attributed to dispersal limitation was 73.4%, indicating that dispersal limitation plays an important role in fungal community assembly [54]. These differences may be related to the specific environmental conditions of the study area. In river and sediment ecosystems, environmental factors (such as pH, temperature, dissolved oxygen, and organic matter content) have a significant impact on the assembly of fungal communities.

### 4.3. Co-Occurrence Network Analysis of Fungal Communities

In addition to the abiotic factors of environmental filtering and dispersal limitation, interspecific interactions are also considered important factors affecting microbial community assembly processes. Network structure can reflect the complexity and stability of communities [55]. Analysis of sedimentary fungal communities across three distinct altitudinal gradients revealed a predominance of positive interspecific associations. This is likely because sediments accepted nutrients that settled from the water, providing conditions for material transfer among fungal community species and facilitating species growth and reproduction through symbiotic or mutualistic relationships [56].

In extreme environments, competition among community species is weakened, and they rely more on mutual cooperation to maintain the river’s ecological network [57]. The average degree and average clustering coefficient were higher in YRA, while the average path length was lower. Moreover, the co-occurrence network of fungal communities in YRA sediments had the most edges, indicating that fungal communities in YRA exhibited more correlations. Thus, the co-occurrence network in YRA was more closely connected, with greater network complexity and connectivity. This phenomenon may be related to the environmental characteristics of high-altitude areas. High-altitude ecosystems impose stringent environmental filters, resulting in selective retention of microbial taxa exhibiting adaptive traits for extremophilic survival and reproductive success. This filtering mechanism drives community homogenization, consequently amplifying interaction frequencies through niche overlap optimization [57].

Network analysis further demonstrated that a more stable network structure was observed in fungal communities at high-altitude areas, which may be attributed to the enhanced adaptive capabilities and stress tolerance exhibited by these fungal species. This stable network structure helps microbial communities better cope with environmental changes and maintain ecosystem functions. For example, certain microbes may enhance the stability and function of the entire community through symbiotic relationships or metabolic complementarity [57,58]. In addition, fungal networks in high-altitude areas have more network nodes, indicating the presence of multiple closely connected subgroups within fungal communities. This structure may reflect the differentiation and synergistic action of fungi in different ecological niches, contributing to the enhancement of the entire ecosystem’s diversity and stability [59].

### 4.4. Main Factors Influencing Fungal Community Changes in Sediments of the Yellow River Basin

As pollutants from the river’s surroundings continue to enter the river environment, they accumulate in the river’s surface sediments, which in turn have a serious impact on the fungal community. In this study, we used redundancy analysis to explore the main environmental factors affecting microbial community differences (Figure 7); Pb, TN, TOC, and silt were the main factors affecting microbial community differences. The impact of environmental factors on microbial community composition may also extend to the transformation of chemical cycling and redox processes; disturbances to microbial community structure often lead to shifts in community function [60]. TOC can affect related microbial community structures, and differences in TOC content can affect microbial community structure, function, diversity, and abundance [61]. Studies have shown that nitrogen addition generally has a positive impact on Ascomycota [62], but the combined addition of nitrogen and phosphorus has a negative impact on Ascomycota [63]. The type and concentration of carbon sources can limit the growth and reproduction of certain fungal groups through environmental filtering. For example, in the study of the middle and lower reaches of the Jialing River, fungal communities at sections with less exogenous material input (such as undisturbed and sand mining disturbed sections) had higher diversity, while those at sections with more exogenous material input (such as tributary-disturbed and engineering-disturbed sections) had lower diversity [64]. Nitrogen addition can limit the growth and reproduction of certain fungal groups through environmental filtering. For example, in the study of the upper reaches of the Yellow River, agricultural land use led to an increase in total nitrogen concentration in the water, and the diversity of fungal communities was significantly reduced [65]. Rivers are open systems, with various substances entering the river environment from the surrounding areas, thereby affecting fungal communities in sediments.

Sediment physicochemical characteristics have been demonstrated to exert profound influences on microbial assemblages [66], with fungal communities exhibiting greater sensitivity to anthropogenic disturbances relative to bacterial counterparts [67], while simultaneously serving as bioindicators of pollution intensity [68]. The impact of particle size characteristics has been shown to significantly influence both microbial abundance and structural diversity within communities [69]. In sedimentary environments, fine silt particles usually have a higher content of mineral salts and can bring microorganisms closer together, allowing them to easily obtain nutrients [70]. Particle size characteristics not only affect microorganisms through chemical properties, but also directly affect microbial life processes through changes in attachable surface area and hydraulic properties [21,71]. In this study, the sediment grain size characteristics of the Yellow River Basin had significant differences. The grain size in the YRA area was mainly silty, while the YRB area was dominated by Cosmid, and the sediment grain size distribution in the YRC area was relatively uniform; this may be due to the topography and hydrodynamic conditions within the basin.

The toxic effects of high heavy metal concentrations are exerted on nearly all microorganisms through the disruption of metabolic functions including protein synthesis [72]. The coexistence of multiple heavy metals has been found to diminish microbial community tolerance, with particularly exacerbated toxicity observed under Cu-Zn co-occurrence conditions [73]. Previous research has demonstrated that niche differentiation and symbiotic network stability constitute key response mechanisms employed by fungal communities in heavy metal-polluted environments [74]. Under heavy metal pollution, niche differentiation is observed in fungal communities, resulting in metal-tolerant fungi emerging as dominant taxa [75]. For example, in mining-affected rivers, fungal communities exhibit higher niche breadth and more stable symbiotic networks, enabling them to better adapt to highly heavy metal-polluted environments [54]. In addition, Pb and Zn are the main factors affecting fungal community structure [72], and our results are similar. Elevated concentrations of copper (Cu) and zinc (Zn) were detected in the YRA area relative to YRB and YRC sampling sites. Furthermore, chromium (Cr), nickel (Ni), and cadmium (Cd) concentrations were consistently elevated in YRA samples. The reduced microbial diversity in YRA is attributed to the cumulative toxicity effects of heavy metal exposure. This also explains why, despite higher contents of carbon, nitrogen, and phosphorus in the YRA area compared to YRB and YRC, the diversity was the lowest. Sediment environmental factors are to some extent drivers of changes in fungal community composition and structure. The unique geographical environment constructs specific habitat patterns of microbial community structure, which provides a theoretical basis for the ecological assessment of microorganisms in the Yellow River Basin and for future research on microbial distribution patterns and community assembly mechanisms.

## 5. Conclusions

A comprehensive analysis of sedimentary fungal communities was conducted in the Yellow River Basin (YRB) aimed at elucidating altitudinal distribution patterns and community assembly mechanisms along the elevation gradient. The fungal communities in the sediments of the Yellow River Basin were mainly composed of Ascomycota, Basidiomycota, Chytridiomycota, Mortierellomycota, and Apicomplexa, with Ascomycota and Basidiomycota being the dominant groups. The diversity (Shannon) of fungal communities decreased gradually with increasing altitude. The distance–decay analysis showed that the environmental distance attenuation effect of fungal communities in sediments at the three altitudinal gradients was stronger than the geographical distance. Environmental factors in sediments play an important role in shaping fungal community structure; TOC, Pb, silt, and TN are the main factors causing differences in fungal community structure. During the assembly of fungal communities, deterministic processes dominate, with the highest contribution from homogeneous selection (69.66%). These findings provide enhanced insights into microbial community assembly mechanisms in aquatic ecosystems, offering critical foundations for developing targeted ecological conservation strategies and sustainable water resource management protocols in the Yellow River Basin.

## Figures and Tables

**Table 1:** Statistical characteristics of fungal community nodes and topological parameters.

Basin	Node	Edge	Average Degree	Average Path Length	Network Diameter	Clustering Coefficient	Density	Heterogeneity	Centralization
YRA	61	328	10.75	2.27	3	0.52	0.18	0.51	0.24
YRB	51	54	2.12	3.13	5	0.21	0.04	0.68	0.08
YRC	45	66	2.93	3.61	5	0.27	0.07	0.63	0.09

**Table 2:** The contributions of sediment factors to changes in fungal communities according to the results of redundancy analysis (RDA).

Basin	Explained Variation (%)			Explanatory Variables (Contribution %)
	Axls 1	Axls 2	All Axes	
YRA	74.84	19.27	94.11	Pb (19.4), Ni (24), TN (17.4), Silty (11.6)
YRB	41.32	12.42	53.74	Ni(13.9), Sand(14.4), TN(10.4)
YRC	96.14	2.17	98.31	TP (27.9), Consimd (24), As (12), NO_3_^−^-N (11.6)
Yellow River Basin	53.26	29.12	82.38	TOC (10.2), Pb (15.5), Silty (10.7), TN (12.3)

## Data Availability

The raw data supporting the conclusions of this article will be made available by the authors on request.

## Supplementary Materials

The following supporting information can be downloaded at https://www.mdpi.com/article/10.3390/jof11030214/s1: Figure S1: Characteristics of sediment carbon, nitrogen, phosphorus, and heavy metal content; Figure S2: Characteristics of sediment grain size distribution; Figure S3: Characteristics of fungal community structure in sediments. Note: The numbers in the figure represent the number of ASVs; Figure S4: Analysis of microbial distance decay in sediments. (A) Relationship between Bray-Curtis dissimilarity and geographical distance in the YRA area. (B) Relationship between Bray-Curtis dissimilarity and geographical distance in the YRB area. (C) Relationship between Bray-Curtis dissimilarity and geographical distance in the YRC area. (D) Relationship between Bray-Curtis dissimilarity and environmental distance in the YRA area. (E) Relationship between Bray-Curtis dissimilarity and environmental distance in the YRB area. (F) Relationship between Bray-Curtis dissimilarity and environmental distance in the YRC area; Table S1: Fungal communities at the phylum level in sediments; Table S2: The top ten non-aquatic fungal communities at the genus level in sediments.
