# Supplementary material for: Distribution Pattern and Assembly Process of Fungal Communities Along Altitude Gradient in Sediments of the Yellow River Basin
Source: J Fungi (Basel). 2025 Mar 11;11(3):214. doi: 10.3390/jof11030214 (PMC11943069; doi:10.3390/jof11030214)
Supplement: Supplementary file 1 [file jof-11-00214-s001.zip › jof-3471736-supplementary.pdf]

1. Materials and methods

1.1 Sample collection and processing

Sediment samples were collected from 44 sampling sites in the Yellow River Basin from July to August 2022, with the main stream sampling sites located at hydrological stations. Approximately 500 g of sediment samples were collected at each sample site using a grab sampler; part of the samples were loaded into 10 mL sterile centrifuge tubes and stored at -80°C for DNA extraction and sequencing; and the other part was stored in sterile sampling bags for subsequent physicochemical analyses. Each sampling point was sampled three times in parallel, and a total of about 1.5 kg of samples were collected from each sampling point. The samples used for physicoche- mical property analysis were air-dried, ground and sieved, and then referred to the national environmental standards of the People's Republic of China for the determination of the relevant physicochemical properties of the sediments, such as total nitrogen (TN), total phosphorus (TP), total organic carbon (TOC) and heavy metals (GB 17378.3-2007).

1.2 Determination of heavy metals in sediments

Multielement calibration standards containing chromium (Cr), nickel (Ni), copper (Cu), zinc (Zn), arsenic (As), cadmium (Cd), and lead (Pb) were prepared in six-point concentration gradients (0, 0.01, 0.02, 0.05, 0.10, 0.20 mg/L) using certified reference materials. Systematic error correction was performed through blank experimentation, with quantitative analysis conducted via hotplate-assisted aqua regia extraction coupled to inductively coupled plasma mass spectrometry (ICP-MS) following EPA Method 3050B. Each sample was processed and filtered through a 0.22 μm microporous filter

membrane, then analyzed on the machine, and the mass concentration of each element was calculated according to the standard curve.

1.3 DNA Extraction and High-Throughput Sequencing

In strict accordance with the manufacturer's specifications, genomic DNA was isolated from sediment samples using a commercial extraction kit, with subsequent quality control performed through 1% agarose gel electrophoresis to verify purity and concentration. The fungal ITS gene was amplified by PCR using the primers. ITS1F (5′-CTTGGTCATTTAG AGGAAGTAA-3′) and ITS2R (5′-GCTGCGTTCTTCATCGATG C-3′), these primers mainly target the ITS1 region for amplification. The PCR amplification conditions for the fungal ITS gene were as follows: the thermal profile comprised (1) initial denaturation at 96℃ (5 min); (2) 35 cycles of denaturation at 96℃ (30 s), annealing at 52℃ (45 s), and extension at 72℃ (1 min); (3) final extension at 72℃ (10 min) [9,25,30].

The raw sequencing data quality was initially assessed using FastQC software, which examined base quality scores, GC content distribution, adapter contamination, and other critical parameters to ensure compliance with the prerequisites for downstream analyses. Data filtration was subsequently performed with Trimmomatic, through which low-quality reads (Phred score < 30), residual adapter sequences, and primer dimers were systematically removed, while high-quality reads were retained for further processing. Finally, a chimera detection pipeline was implemented using DADA2, enabling the identification and elimination of artifactual chimeric sequences generated during PCR amplification cycles, thereby ensuring the biological validity of the analytical results. Operational taxonomic unit (OTU) clustering was subsequently performed using QIIME2, with sequences sharing ≥97%

similarity threshold being grouped into discrete clusters serving as proxies for microbial species delineation. To improve resolution, we also used DADA2 software for ASV clustering to identify unique sequence variants in each sample. Subsequently, BLAST analysis was used to compare the representative sequences of ASVs with known sequences in the NCBI database. Based on the comparison results, the taxonomic status of each ASV was determined, including domain, phylum, class, order, family, genus, and species.

2. Figures

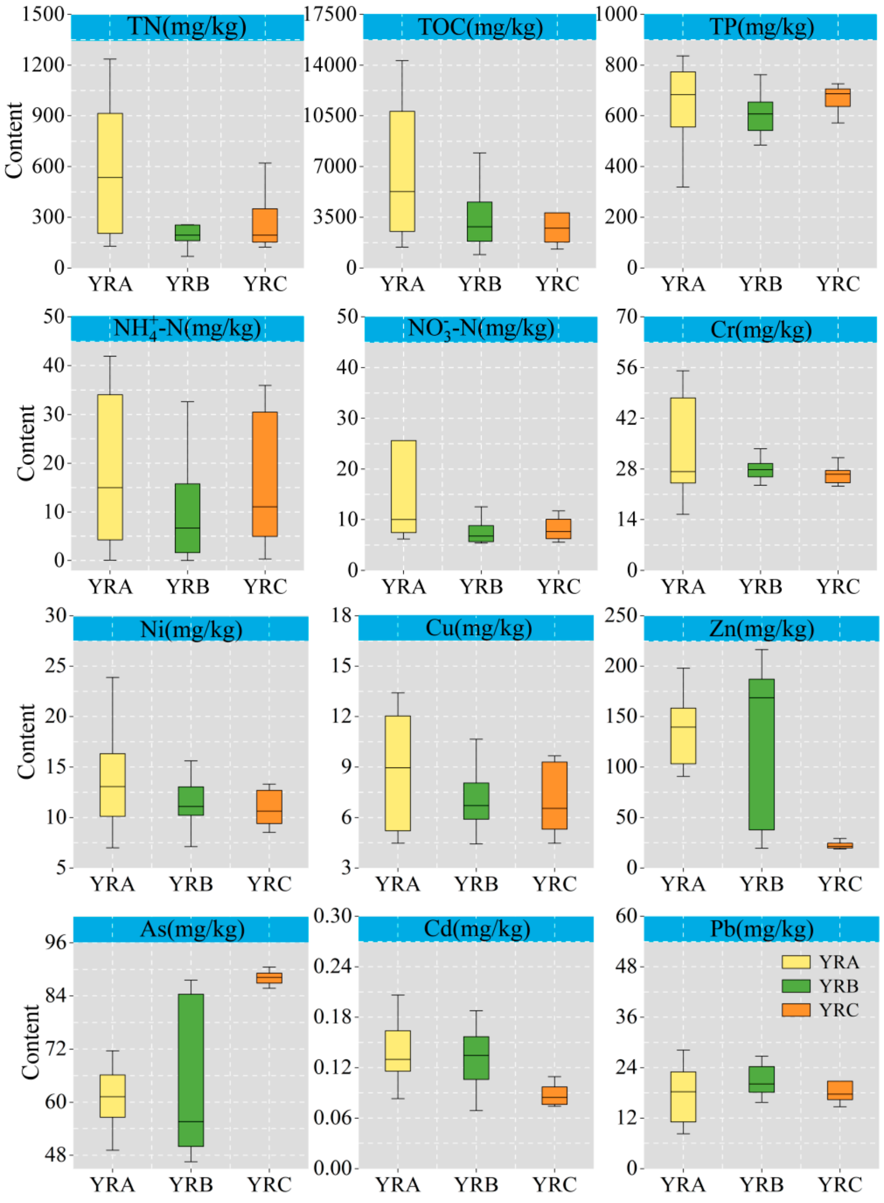

Figure S1. Characteristics of sediment carbon, nitrogen, phosphorus, and heavy metal content.

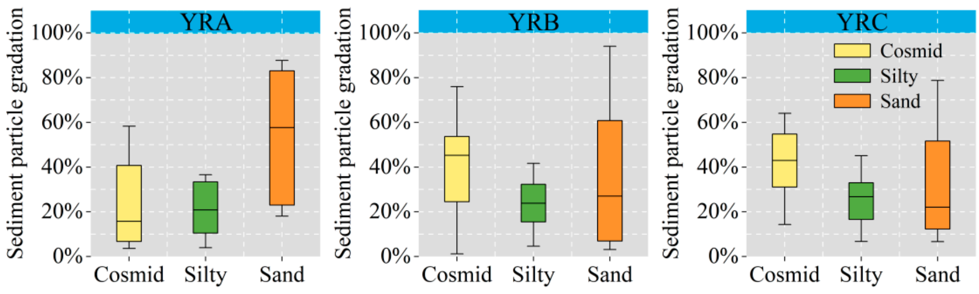

Figure S2. Characteristics of sediment grain size distribution.

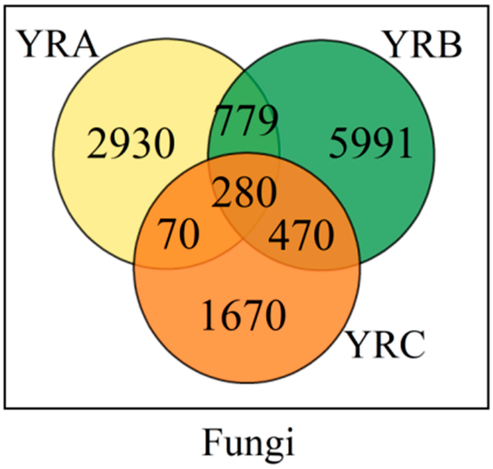

Figure S3. Characteristics of fungal community structure in sediments. Note: The numbers in the figure represent the number of ASVs.

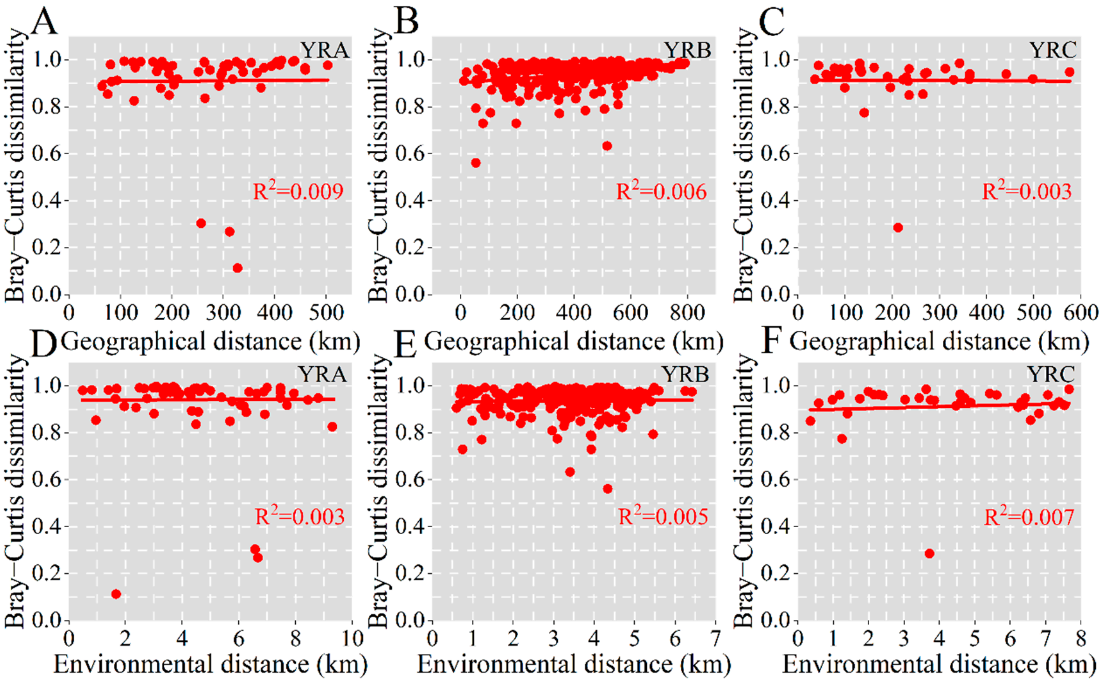

Figure S4. Analysis of microbial distance decay in sediments. (A) Relationship between Bray-Curtis dissimilarity and geographical distance in the YRA area. (B) Relationship between Bray-Curtis dissimilarity and geographical distance in the YRB area. (C) Relationship between Bray-Curtis dissimilarity and geographical distance in the YRC area. (D) Relationship between Bray-Curtis dissimilarity and environmental distance in the YRA area. (E) Relationship between Bray-Curtis dissimilarity and environmental distance in the YRB area. (F) Relationship between Bray-Curtis dissimilarity and environmental distance in the YRC area.

3. Tables

Table S1. Fungal communities at the phylum level in sediments.

Basin	Ascomycota	Unclassified	Basidiomycota	Chytridiomycota	Mortierellomycota	Neocallimastigomycota	Glomeromycota	Olpidiomycota	Blastocladiomycota	Other
YRA	58.53%	18.25%	11.17%	2.48%	4.16%	4.59%	0.09%	0.30%	0.00%	0.44%
YRB	38.64%	26.89%	18.96%	9.10%	2.54%	0.17%	0.23%	0.11%	0.04%	3.32%
YRC	57.61%	21.08%	12.44%	4.30%	2.12%	0.00%	0.30%	0.01%	0.00%	2.14%

Table S2. The top ten non-aquatic fungal communities at the genus level in sediments.

Basin	Fusarium	Coprinellus	Mortierella	Trichoderma	Gibberella	Subulicystidium	Pseudeurotium	Preussia	Unclassified	Other
YRA	23.88%	0.14%	4.08%	0.28%	0.46%	0.00%	3.82%	2.57%	30.32%	34.44%
YRB	2.56%	3.14%	2.50%	4.42%	4.46%	2.84%	0.34%	1.24%	52.73%	25.77%
YRC	23.13%	6.03%	2.12%	1.50%	0.36%	0.00%	1.40%	0.33%	35.74%	29.39%
